# Reconstruction intramedullary nailing for a failed subtrochanteric Seinsheimer type IIB fracture: a case report

**DOI:** 10.3389/fsurg.2023.1172971

**Published:** 2023-05-12

**Authors:** Fei Wang, Tianfeng Liu, Shoujin Guo, Lei Wu, Peiwang Xin

**Affiliations:** Orthopedics, Feicheng Traditional Chinese Medicine Hospital, Feicheng, Tai'an, China

**Keywords:** subtrochanteric fracture of femur, femoral reconstruction intramedullary needle, Seinsheimer type IIB fracture, nail off, fail

## Abstract

**Introduction:**

A case of subtrochanteric Seinsheimer II B fracture was retrospectively analyzed to determine the causes of failure and the possible problems with femoral reconstruction intramedullary nailing.

**Methods:**

This study focused on a case of an elderly patient with Seinsheimer type IIB fracture treated with minimally invasive femoral reconstruction intramedullary nailing. By retrospectively analyzing the intraoperative and postoperative course, we can identify the reasons that may lead to the surgical failure in order to avoid similar problems in the future.

**Result:**

It was observed that the nail was dislodged after surgery, and the broken end was displaced again. Through our analysis and research, we believe that non-anatomical reduction, deviation of needle insertion point, improper selection of surgical methods, mechanical and biomechanical effects, doctor–patient communication and non-die-cutting cooperation, and non-compliance with doctor's orders may be related to the success of surgery.

**Conclusion:**

Femoral reconstruction intramedullary nailing is used for the treatment of subtrochanteric Seinsheimer II B fractures; however, non-anatomical reduction, choice of needle insertion point, inappropriate choice of surgical method, mechanical and biomechanical effects, doctor–patient communication and cooperation without die-cutting, and non-compliance with doctor's advice may result in surgical failure. According to the analysis of individuals, under the premise of an accurate needle entry point, minimally invasive closed reduction PFNA or open reduction of broken ends and intramedullary nail ligation for femoral reconstruction can be used in Seinsheimer type IIB fractures. It can effectively avoid the instability of reduction and the insufficiency of the biomechanics caused by osteoporosis.

## 1. Introduction

It is generally believed that a subtrochanteric fracture of the femur refers to the fractures between the upper edge of the small rough dragon and the site of femoral stenosis (1). Sometimes, the proximal end of the fracture continues to the large rotor, and the distal small rotor extends down the 5-cm range of the fractures (4, 6–8, 15, 16, 18). Subtrochanteric fracture of the femur accounts for approximately 10%–30% of hip fractures (4, 5, 8). In young people, it occurs mainly due to high-energy trauma, whereas in older persons, it is considered to be related to pathological changes or osteoporosis (2, 8). Subtrochanteric fractures of the femur are difficult to reduce and stabilize due to their special biomechanics, unique anatomy, and concentrated load stress (2, 6, 16, 22). Regarding the management of subtrochanteric fracture of the femur, most of the literature recommends surgical treatment (3, 18), which can avoid the numerous disadvantages of conservative treatment; however, the choice of the surgical method is still controversial (1, 17, 18). Herein, we analyzed the reasons for surgical failure and the possible problems of a case of subtrochanteric Seinsheimer IIB fracture treated by femoral reconstruction intramedullary nailing.

## 2. Materials and methods

### 2.1. Patient

The patient was a seventy-year-old female with a subtrochanteric helical fracture of the femur due to a sprain from an accidental fall downstairs (torsion violence), who had a history of rheumatoid arthritis for over 10 years, which was controlled by self-medication. The patient's BMI (Chinese standard) was 45/(1.65) 2≈16.53 < 18.4, thin. No other basic diseases and family genetic history were recorded.

### 2.2. Diagnosis

**Figure 1:**
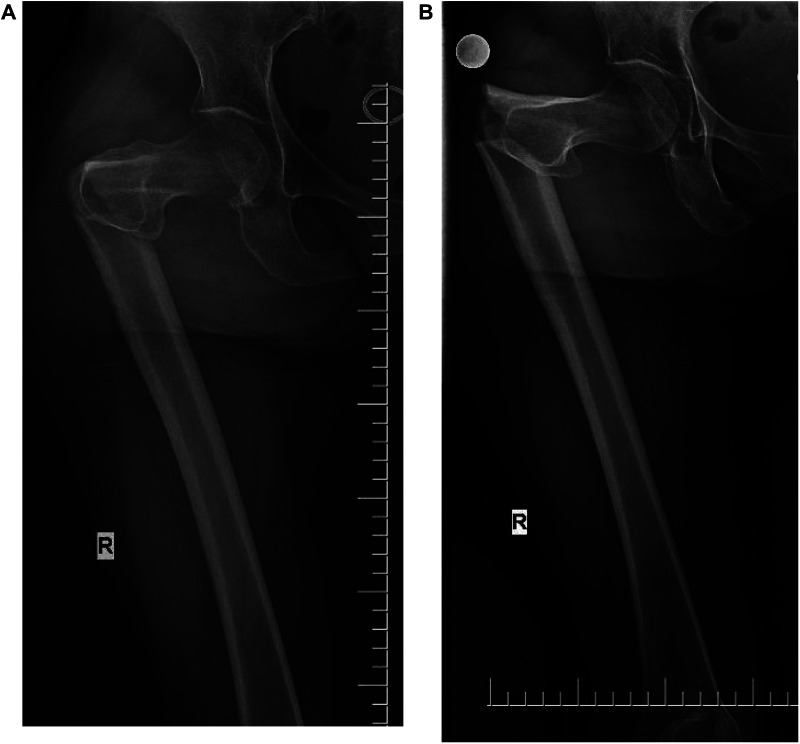
(**A**) Anterior radiograph fracture with angular deformity. (**B**) The fracture was significantly displaced, with proximal posterior rotation.

Anteroposterior and lateral radiographs of the femur showed a subtrochanteric spiral fracture of the femur with proximal posterior rotation, separation, and displacement of the broken ends and angulation (Figures 1A,B). The patient presented with tenderness and percussion pain along the longitudinal axis of the right lower extremity, as well as flexion shortening and valgus deformity of the right lower extremity. The right hip was obviously painful and swollen, and the functional activities of the right lower extremity were limited. The fractured part was similar in morphology to a “pseudo-joint.” The patient was positive for percussion pain on the longitudinal axis of the right lower limb. Subtrochanteric Seinsheimer type IIC fracture refers to a distal fracture of the small rotor. The two can be distinguished by x-ray and CT (Computed Tomography).

### 2.3. Surgical process

The patient was admitted to the hospital, and relevant examinations were conducted to exclude any contraindications of surgery. After reading the radiographic images, the surgeon decided to perform a minimally invasive incision treatment using a femoral reconstruction intramedullary nail with the consent of the patient and their family. After successful combined spinal-epidural anesthesia, the patient was placed in the supine position, bed traction of the affected limb was performed, the healthy limb was placed in the lithotomy position, the perineal blocking pad was placed in the groin of the unaffected side—with buttocks of the affected side being placed are as far away as possible—the bed traction was externally rotated under C-arm fluoroscopy, and abduction, manual squeezing, lifting, and pressing were also performed to reset the broken end (three points and one line: the midpoint of the groin–knee–ankle point was positioned on a straight line, and the toes were vertically upturned). After fluoroscopic observation of the alignment, the surgical area was routinely disinfected and covered with sterile towels and a brain surgical skin film. The anatomical projection of the apex of the greater trochanter was determined by hand touch, and an incision of approximately 5 cm was made obliquely from the proximal end to the distal end to the apex of the greater trochanter. Separation forceps were used to separate the surrounding tissues, the fascia was opened longitudinally, and the apex of the greater trochanter was touched. After the needle point was determined by fluoroscopy under the C-arm machine, with the aid of the sleeve, an opener was inserted obliquely at a 45° angle in the direction of the needle to the position of the minor trochanter. After completion, the long guide needle was inserted into the distal end, and the implantation and broken end of the guide needle was observed under the C-arm machine. An electric drill was used to expand the proximal medullary cavity, and then a 6.5-mm drill bit was used to expand the distal medullary cavity. According to the condition of the patient's medullary cavity, the distal medullary cavity was increased by 1 mm. Under the C-arm machine, the intramedullary needle implantation and broken ends were seen by fluoroscopy, and two locking nails were implanted along the distal end of the guide. The intramedullary needle was knocked back to shorten the separation between the broken ends, and the proximal double half-threaded screw was driven along the guide according to the perspective of the C-arm machine, which was satisfactory. After satisfactory surgery, rinse, suture, and bandage the wound. Remove the anesthesia tube and return to the ward.

After the operation, antibiotic treatment was given for 48 h, and intervention treatment for anti-osteoporosis and promoting fracture healing was performed. The patient could stay in bed for 14 days and perform simple standing activities with the aid of an auxiliary device. One month after surgery, simple walking activities could be performed with the aid of an auxiliary device. After three months of surgery, the patient could gradually disengage from the auxiliary tool for autonomous activities. During this period, the patient avoided sitting for a long time to prevent nail peeling during nail cutting. The patient was instructed to undergo a routine X-ray examination at two month after surgery to assess fracture healing, hip joint morphology, and implant status. The patient and their family members did not undergo routine medical examinations as instructed. After two months of surgery, the patient consciously contacted the foreign body in the hip to receive reexamination.

## 3. Result

**Figure 2:**
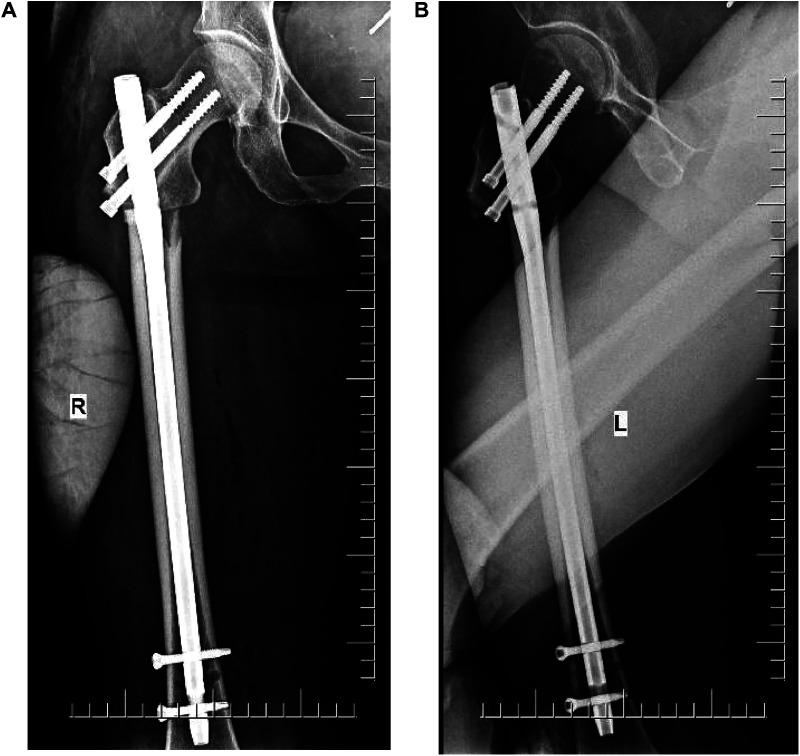
(**A,B**) X-ray (4 days after surgery). The lateral end of the fragment shows a displacement.

**Figure 3:**
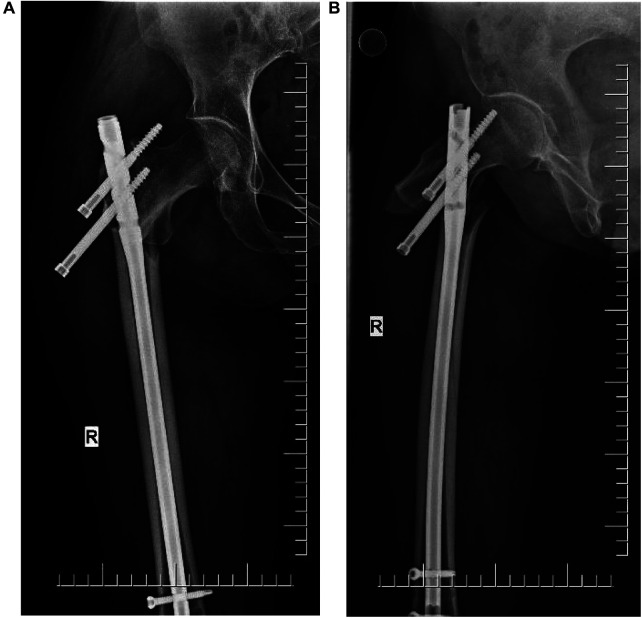
(**A,B**) X-ray (2 months after surgery). The proximal end of the nail was dislodged, and the broken end was displaced again.

Radiographs were conducted four days after surgery (Figures 2A,B). The affected hip could be flexed and extended two months after the surgery. Self-reported hip pain was obvious, which worsened after activity, and screws and bone-like objects were palpable on the outer side of the proximal thigh. The radiographic images showed that the proximal double half-threaded screw and the proximal end of the intramedullary needle were prolapsed, and the broken ends were separated and displaced again. There was no obvious callus formation around the fracture zone (Figures 3A,B).

## 4. Discussion

The incidence of Subtrochanteric fracture of the femur is approximately 25% among the elderly (12). Such fractures are mostly affected by factors such as osteoporosis and their own anatomical structure characteristics, biomechanics, and blood supply. Femoral fractures are caused by a slight external force (2, 6–8, 22). The spiral destruction of the medial cortex and the incompleteness of the lateral cortex make reduction and maintenance difficult, and there is a risk of internal fixation failure or non-union (7, 22, 25), as well as difficulties and complications in treatment and rehabilitation (18). Surgery includes intramedullary, extramedullary, and external fixation (3, 4, 6, 7, 12, 22, 25). At present, intramedullary treatment is widely recognized and recommended based on its minimal invasiveness and reliable biological stability, especially in elderly patients (3, 4, 6, 7, 12, 15–18, 22, 25). The Seinsheimer IIB fracture is a spiral fracture in which the lesser trochanter is located proximal end of the fracture.

## 5. Causes of failure and possible problems

### 5.1. Spiral fracture and instability

Intraoperative reduction and separation, the limitation of traction maintenance, intraoperative reaming, and handle needle insertion are prone to fracture reduction deviation and implant failure. Older persons, poor constitution, and osteoporosis lead to insufficient nail stability and holding force, and cortical integrity damage leads to poor stability. The subtrochanteric area is the stress concentration area. From the perspective of biomechanics and anatomical structure, many scholars believe that the anatomical reduction of a subtrochanteric fracture of the femur, the integrity of the medial and lateral cortex, and the stability of the broken ends are very important for the success of surgery and the promotion of fracture healing (2, 4, 6–8, 11, 18, 22). The fracture site is dominated by cortical bone, and the stress and stability of the medial cortical spiral rupture are weakened. It is affected by the contraction and traction of the distal and proximal muscle groups and the blood supply, resulting in difficult reduction and slow healing (4, 8, 11, 22). Seinsheimer IIB fractures are unstable fractures (36). Considering older patients, fractures with simple spiral displacement without free fragment and small incision, the advantages of the proximal double-tension semi-threaded screw for reconstruction, and the wall thickness of the proximal intramedullary needle, the fracture resistance to bending and shear force is enhanced. The femoral reconstruction intramedullary needle was selected because the wound incision is less destructive to the surrounding tissue and blood supply, and it is beneficial to fracture healing and early functional activity after good reduction (1). However, the patient's advanced age, poor physical condition, and osteoporosis, as well as the damage of the lateral cortex and the rupture of the medial cortex during the surgery, weakened the resistance and holding force of the proximal screw and led to tension screw removal under the influence of the biomechanics after surgery. In addition, flexion and extension joint activity and cortical cutting may also lead to the problem of screw off. Small incisions cannot directly visualize the integrity of the cortical bone. It cannot be ruled out that the lateral cortex was damaged during the reaming and proximal screw tunnel construction. These can lead to biomechanical instability, resulting in surgical failure.

### 5.2. No anatomical reduction

**Figure 4:**
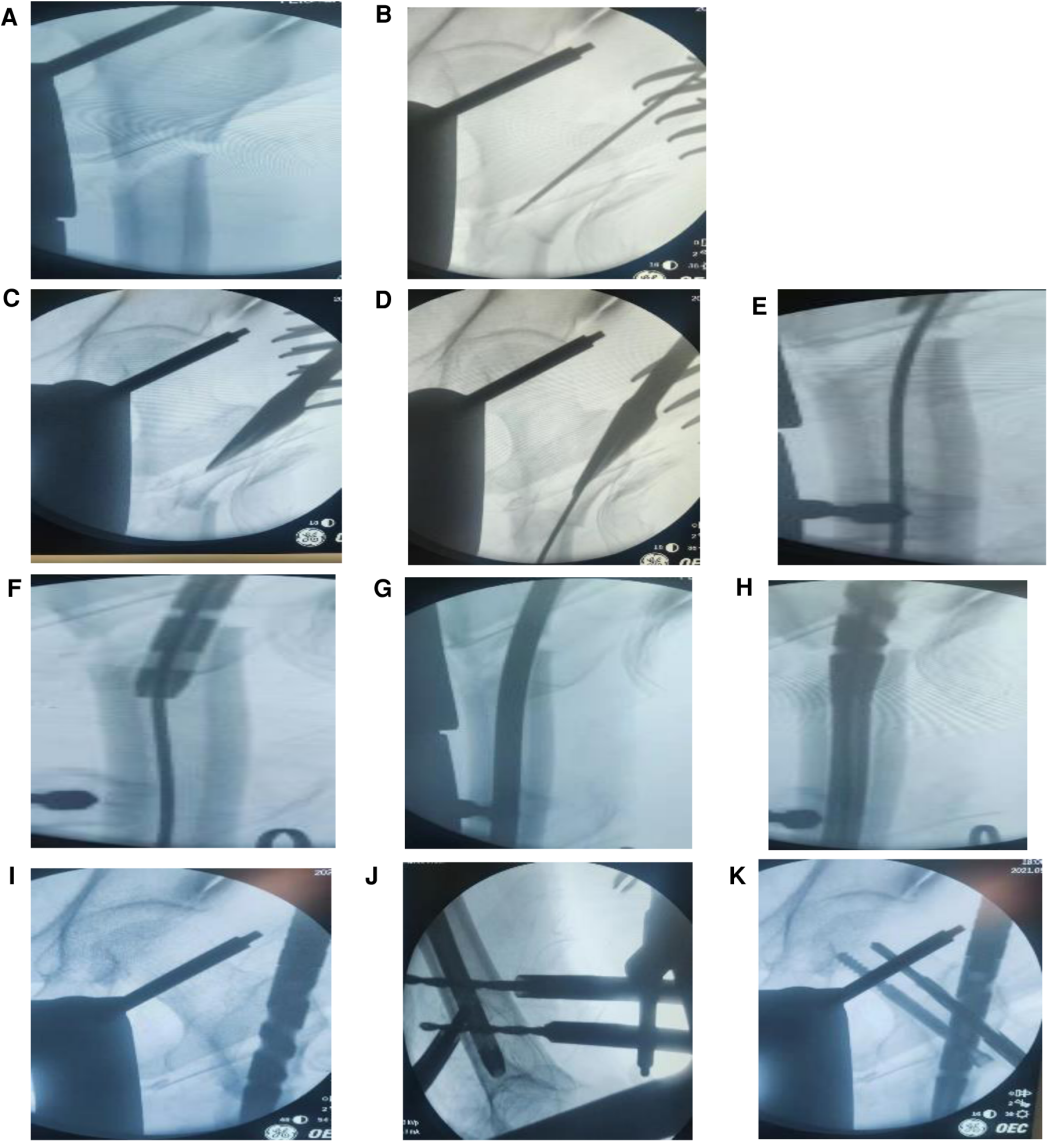
(**A–K**) The whole process of surgical traction reduction, needle threading, and nailing.

Anatomical reduction of fractures is a key factor in the success of surgery (2, 4, 7, 12, 15). In intramedullary minimally invasive fixation, there is a blind spot in reduction, and familiarity with anatomy and skilled manipulation are particularly important at this time. Anatomical reduction of fractures and maintenance of alignment after reduction are very important for intramedullary fixation. The surgery was performed in a supine position, with bed traction, reduction, and fixation of the affected limb and correction of the neck-shaft angle and anteversion angle. Due to the patient's spiral fracture, fracture separation into right-angle deformity, medial cortical rupture, simple alignment of the traction bed, contraposition support (there are unstable factors in the separation between the two points and one line), and the impact of the distal and proximal anatomical muscles after reduction, reset was extremely unstable. Intraoperative images showed the separation of broken ends, which further increased the instability of the fracture after reduction, and it was difficult to maintain the vertical line. During the implantation of intramedullary needles, there is a risk of proximal deviation and angular displacement due to the guiding force of the needle and the pulling of the muscle. Therefore, skilled needle threading techniques and surgical skills are particularly important at this time (1) (Figure 4A).

**Figure 5:**
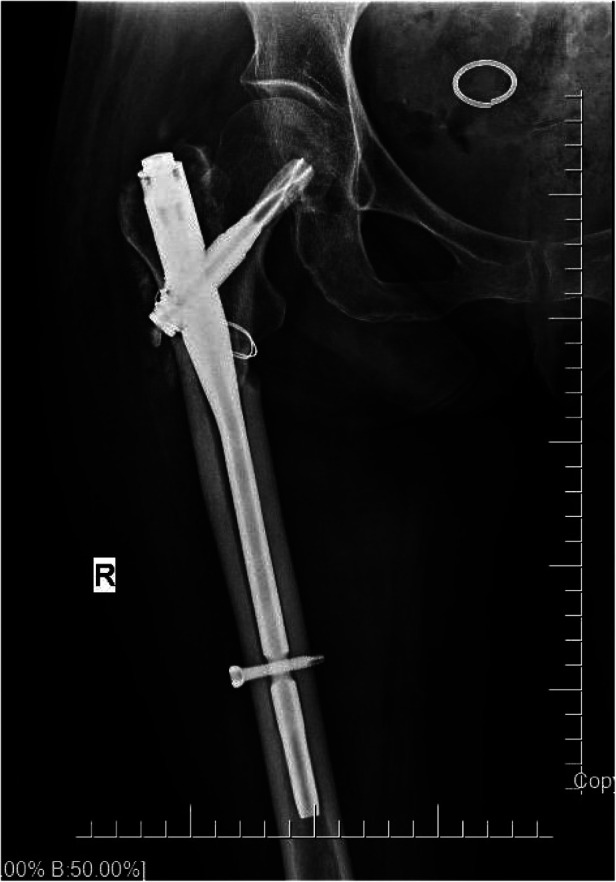
After repair, PFNA and steel wire were used for internal fixation.

### 5.3. The entry point and opening position of the nail are on the outside, the medullary cortex ruptures, and the intramedullary needle slides (Figure 4B-D)

It is mostly recommended that the needle insertion point should be located at the apex of the greater trochanter and the fossa ovalis (14). After the needle insertion point deviates slightly outside the apex of the greater trochanter, the intramedullary needle sticks to the proximal medial wall during the needle insertion process. Therefore, the needle insertion process becomes difficult, and the fracture is displaced, or the proximal cortex is penetrated during the forced needle insertion process. The weak part is freed from the outside of the medullary cavity, the intramedullary needle is drawn into the distal medullary cavity, or the posterior cortex is destroyed after the proximal reaming, which causes the intramedullary needle to stick to the posterior cortex, squeeze the proximal anterior side, and move into the distal medullary cavity. In addition, when the electric drill bit at the needle entry point was used to expand the proximal medullary cavity for the first time, the positioning point was displaced posteriorly due to the instability of the positioning needle or the rupture of the outer and posterior cortex. The intramedullary fixation failed due to the crushing damage to the bone cortex and the incomplete cortex itself during the entry of the electric drill. The patient was old, had been taking rheumatoid arthritis drugs over a long period, had a poor nutritional status, and had obvious osteoporosis. There was a risk that the intramedullary needle could deviate from the normal medullary cavity and break the cortex during the needle insertion process. Reaming of the medullary cavity leads to weak cortical strengthening or rupture and increases the risk of dislodgement or penetration of the nail out of the medullary cavity. The intramedullary needle is implanted according to the reduction situation and the fracture vertical line adjustment handle to ensure a satisfactory anatomical angle (Figures 4B–D).

### 5.4. The absence of lateral radiographs cannot determine whether the intramedullary pin is in the medullary cavity and the reduction of the fracture

Intraoperative minimally invasive intramedullary fixation or intramedullary needle implantation has a blind spot. In addition, the fracture is unstable, or the cortical bone is damaged, and the distal and proximal fracture ends are likely to be separated again during the slippage of the implantation point and the needle threading process. The proximal intramedullary needle slides from the posterior wall into the distal intramedullary needle and is implanted into the medullary cavity, resulting in a false impression of good implantation, and x-ray fluoroscopy shows a false good reduction and implantation (Figures 4E–I). Therefore, a perfect frontal and lateral radiograph is particularly important at this time to locate the intramedullary needle and assess the fracture (14).

### 5.5. The operator's proficiency, psychological quality, and lack of prospective assumptions and potential problems before surgery

Bed traction combined with manual closed reduction of the fractured ends requires high surgical proficiency and surgical skills on the part of the operator and assistant. Strong basic knowledge and profound clinical practice experience are critical to the success of surgery. Physicians are kind and always maintain a humble attitude and the concept of being responsible to patients. An impetuous mood and surgical self-satisfaction strongly influence judgment. However, there is a lack of comprehensive surgical evaluation to predict potential problems and systematic postoperative protection.

### 5.6. There is a lack of confirmatory examinations after surgery

Postoperative hip flexion and knee flexion check to determine the success of intramedullary pinning. This treatment lacks a sure-fire test, and there is no difference in length and varus deformity of the lower extremities.

### 5.7. Physical influence

The patient was old, had long-standing history of rheumatoid arthritis, and had obvious osteoporosis. There is a possibility of postoperative screw loosening and screw removal due to shear tension, anatomical and biological stress, or improper limb function exercise and transfer.

### 5.8. Improper postoperative care and handling measures

After the surgery, the affected limbs were guided by the doctor and the family members to follow the body's center of gravity in order to move in parallel, turn over, flex, and extend the limbs. When lying on the side, the center of the knee joints was suitable for materials and highly supported for the affected limbs, and the back was supported by the family members to stabilize the body balance. Violently turning over or carrying, improper movement of the affected limb or an unstable body center of gravity, sudden collapse of the body or natural sagging of the affected limb, placing the affected limb on top during lateral recumbency, and gravity or torsional stress may cause the screws to cut the cortical bone or nails.

### 5.9. Violation of doctor's orders

Patients should rest in bed for one week after the surgery, after which they can do sit–stand training. When the patient has a concealed behavior, emotionally dominates the movement of the affected limb after returning home, or sits down for a long time, this could result in screw cutting and loosening.

### 5.10. Poor doctor–patient communication

Poor doctor–patient communication involves a lack of good understanding between the doctor and the patient, misunderstanding in the exchanges between them, and poor implementation of the doctor's orders.

There are many controversies between open reduction and closed reduction for subtrochanteric fractures of the femur. There is no significant difference between the two in terms of postoperative fracture healing, superficial tissue infection, and related complications (1, 8). Clinical failure of the intramedullary fixation of the unstable subtrochanteric fracture of the femur is common (8–10, 25), with a failure rate of 1.9% (23). Both intramedullary and extramedullary fixation were available for this patient (8). However, we think that intramedullary fixation causes less tissue damage, has less blood supply images, and leads to better biological stress, especially in elderly patients (13, 22). There was no significant difference in postoperative rehabilitation and complications between intramedullary and extramedullary fixation (26). Proficient surgical skills, lightweight surgical skills, precise needle entry point positioning, traction anatomical alignment stability, and anatomical reduction are important factors for intramedullary fixation (2, 4, 11). Under the premise of many factors, minimally invasive intramedullary fixation still has a high success rate. Whether it is proximal femoral nail antirotation (PFNA) or reconstruction nailing, it is a good choice. The pure intramedullary fixation of PFNA appears to be more stable with the aid of a helical blade, but this is just an assumption. Reconstruction of the intramedullary needle may be considered as an anatomical reduction of fracture clamping, and ligation of the broken end by wire or titanium cerclage is more conducive to the stability of the broken end and the success rate of the surgery. It has been reported that ring binding can destroy the local blood supply or periosteum, and ring rod rolling may cause necrosis or non-union (4, 27), but there is no definite confirmation (28). Through biostress studies, Muller T (29). and others believed that ring binding is beneficial for proximal screws, biomechanics, fracture stability, reduction, and surgical success (8, 11). It has little to do with periosteal destruction and blood supply imaging (18–20) and is conducive for recovery and risk aversion (21).

Temporary reduction and stabilization of fractures were performed with steel plates, and satisfactory results were obtained in the case of difficult reduction (15, 24, 30). There was no significant difference in postoperative recovery between open and non-open reduction (4). The outcome of intramedullary fixation is uncertain, and effective external fixation is necessary (2, 27). Intramedullary fixation of subtrochanteric fractures has a risk of failure or non-union (31). It has its own advantages and disadvantages in terms of the choice of the surgical position and the needle entry point in that it could be performed while lying on the side or in prone or supine position (6, 14, 16). The best choice can be made according to the fracture situation, intraoperative needs, and familiarity, and the needle insertion point can be grasped. The latest view is that the prone position has advantages in subtrochanteric fractures (16). They are critical for anatomical reduction, internal fixation stability, biological stability, and accurate needle entry points, as well as to ensure satisfactory neck-shaft and anteversion angles. Due to the advantages of intramedullary fixation in terms of biomechanical stability and minimal soft tissue damage, currently, most patients choose intramedullary fixation for the treatment of such fractures (2). However, most studies have not yet confirmed that intramedullary fixation is superior to extramedullary fixation (1).

Subtrochanteric fractures of the femur are unstable femoral fractures, which pose a risk of delayed union and non-union after treatment (32). There is currently no definitive treatment for implant rupture caused by delayed healing and non-union (33). Practitioners should generally follow moderate recommendations for the treatment of patients with unstable intertrochanteric fractures using a cephalomedullary device (34). Anti-osteoporosis treatment in elderly patients is also crucial. A patient with subtrochanteric fracture and implant rupture was treated with internal fixation with a long femoral nail. The implant was removed, and total hip arthroplasty was performed through a posterior approach, with satisfactory late results (35). In addition, the use of grooved tapered modular stems with distal fixation has potential advantages in femoral revision and post-traumatic situations (36). In terms of osteoporosis, patients were instructed to take calcium carbonate D3 for anti-osteoporosis treatment after surgery, without any other relevant intervention treatment.

In conclusion, there is a possibility of surgical failure of the treatment of subtrochanteric Seinsheimer IIB fractures through various means, such as no anatomical reduction, deviation of the selected needle insertion point, selection of inappropriate surgical methods, mechanical and biomechanical effects, incompatibility of doctor–patient communication and cooperation, and non-doctor-ordered behaviors. According to the analysis of individuals, under the premise of an accurate needle entry point, minimally invasive closed reduction PFNA or open reduction of broken ends and intramedullary nail ligation for femoral reconstruction can be used in Seinsheimer type IIB fractures, which can effectively avoid the instability of reduction and the insufficiency of the biomechanics caused by osteoporosis. For clinical reference.

## Data Availability

The original contributions presented in the study are included in the article, further inquiries can be directed to the corresponding author.
